# Vascular Notch Signaling in Stress Hematopoiesis

**DOI:** 10.3389/fcell.2020.606448

**Published:** 2021-01-21

**Authors:** Can Huang, Dawei Yang, George W. Ye, Charles A. Powell, Peipei Guo

**Affiliations:** ^1^McCann Health Medical Communications, New York, NY, United States; ^2^Zhongshan Hospital Fudan University, Zhongshan Hospital Institute for Clinical Science, Shanghai Medical College, Fudan University; Shanghai Engineering Research Center of AI Technology for Cardiopulmonary Disease, Shanghai, China; ^3^Division of Pulmonary, Critical Care, and Sleep Medicine, Fibrosis Research Center, Icahn School of Medicine at Mount Sinai, Mount Sinai-National Jewish Respiratory Institute, New York, NY, United States

**Keywords:** endothelial cells, notch signaling, stress hematopoiesis, T cell immunity, tumor

## Abstract

Canonical Notch signaling is one of the most conserved signaling cascades. It regulates cell proliferation, cell differentiation, and cell fate maintenance in a variety of biological systems during development and cancer (Fortini, 2009; Kopan and Ilagan, 2009; Andersson et al., 2011; Ntziachristos et al., 2014). For the hematopoietic system, during embryonic development, Notch1 is essential for the emergence of hematopoietic stem cells (HSCs) at the aorta-gornado-mesonephro regions of the dorsal aorta. At adult stage, Notch receptors and Notch targets are expressed at different levels in diverse hematopoietic cell types and influence lineage choices. For example, Notch specifies T cell lineage over B cells. However, there has been a long-lasting debate on whether Notch signaling is required for the maintenance of adult HSCs, utilizing transgenic animals inactivating different components of the Notch signaling pathway in HSCs or niche cells. The aims of the current mini-review are to summarize the evidence that disapproves or supports such hypothesis and point at imperative questions waiting to be addressed; hence, some of the seemingly contradictory findings could be reconciled. We need to better delineate the Notch signaling events using biochemical assays to identify direct Notch targets within HSCs or niche cells in specific biological context. More importantly, we call for more elaborate studies that pertain to whether niche cell type (vascular endothelial cells or other stromal cell)-specific Notch ligands regulate the differentiation of T cells in solid tumors during the progression of T-lymphoblastic lymphoma (T-ALL) or chronic myelomonocytic leukemia (CMML). We believe that the investigation of vascular endothelial cells' or other stromal cell types' interaction with hematopoietic cells during homeostasis and stress can offer insights toward specific and effective Notch-related therapeutics.

## Introduction

Adult hematopoietic stem cells (HSCs) reside at the apex of hierarchical lineage trees and give rise to life-long supplies of blood and immune system cells. The tight regulation of adult HSC homeostasis and regeneration is orchestrated by the intricate signaling crosstalk between HSCs and non-hematopoietic niche cells in the bone marrow (BM) microenvironment (Crane et al., 2017; Lucas, 2017). Evolutionarily conserved, Notch mediates the paracrine communication between neighboring cells of the same type or different types *via* the direct engagement of ligands and receptors. Notch signaling has been shown to specify the differentiation of T cells or megakaryocyte lineages (Radtke et al., 1999; Han et al., 2002; Mercher et al., 2008) and is dispensable for the maintenance of adult HSCs or differentiation into myelo-erythroid lineages at homeostatic or regenerative scenarios (Maillard et al., 2008; Duarte et al., 2018; Maillard and Pear, 2018). On the other hand, Notch2 promotes HSC regeneration after myelosuppression (Varnum-Finney et al., 2011). Vascular endothelial cells express Jagged1 and Jagged2 that are essential for regenerative hematopoiesis (Poulos et al., 2013; Guo et al., 2017). Endothelial or other BM microenvironment-Notch signaling prevents HSCs from aging and the development of myeloproliferative disease (Wang L. et al., 2014; Kusumbe et al., 2016; Shao et al., 2019; Vanderbeck and Maillard, 2019), highlighting the cell non-autonomous role of Notch in HSC maintenance.

The aim of this mini-review is to make sense of the conflicting observations by comparing the different *in vivo* and *in vitro* models used to study the Notch signaling pathway (Table 1). We shall distinguish the roles of cell autonomous or cell non-autonomous Notch signaling in regulating HSC function. We can also benefit from delineation of the Notch downstream signaling events within HSCs or endothelial cells themselves in the event of endothelial cell-specific Notch ligand deletion. As Notch signaling specifies HSC to T cell differentiation and is oftentimes mutated in hematopoietic malignancies, we have also included a brief discussion on whether niche cells (endothelial cells or stromal cells) disrupt the presentation of their Notch ligands to mis-regulate T cell function, and modulate the disease progression of hematopoietic malignancies. Pan-notch inhibitor gamma secretase inhibitor failed in clinical trials due to on-target gut toxicity (Palomero and Ferrando, 2009; Aster and Blacklow, 2012). We hope that this review can shed light on the urgency in identifying the niche-derived Notch signaling for HSC regeneration or hematopoietic malignancies, which will guide the design of specific and effective Notch-based therapeutics.

**Table 1:** References summarizing the experimental models to dissect the role of Notch signaling in stress hematopoiesis.

**Study model**	**Target cells**	**Notch target**	**Role/impact**	**References**
**NOTCH SIGNALING IN HSC MAINTENANCE, REGENERATION AND DIFFERENTIATION**				
Mx1-cre; Notch1fl/fl	Hematopoietic cells and stromal cells	Not reported	Reduced thymus size and morphology; developmental arrest at double negative T cell stage; increased B cell numbers in thymus	Radtke et al., 1999
Mx1-cre; RBPJfl/fl	Hematopoietic cells and stromal cells	Not reported	Block of T cell differentiation at the earliest stage with an increase of B cells in the thymus	Han et al., 2002
OP9-DL1-HSC coculture; dnMAML1-HSC	Hematopoietic cells	Hes1, Pu.1, Fli1, Gata1	OP9-DL1 promoted HSC differentiation into megakaryocytes; RBPJ/ICN/MAML Complex Mediates Megakaryocyte Development	Mercher et al., 2008
Mx1-cre; RBPJfl/fl; Mx1-cre; Rosa26 dnMAML1	Hematopoietic cells and stromal cells	Hes1, Dtx1	Stable engraftment of dnMAML transduced HSCs; Reduction of T cell differentiation of the dnMAML transduced HSCs	Maillard et al., 2008
Mx1-cre; RBPJ mice; Vav1-cre; RBPJ mice	Hematopoietic cells and stromal cells	Hes1, Hes5, Nrarp, Gata3	Canonical Notch signaling is dispensable for all investigated stages of megakaryocyte, erythroid, and myeloid progenitors	Duarte et al., 2018
Mx1-cre; Notch1fl/fl mice; Mx1-cre: Notch2fl/fl mice	Hematopoietic cells and stromal cells	Hes1	Notch2 but not Notch1 inhibit HSC myeloid differentiation on immobilized Notch ligands; Notch2 but not Notch1 governs regeneration of LT-HSCs	Varnum-Finney et al., 2011
VEcad-cre; Jag1fl/fl mice	Endothelial cells	Hes, Hey1	Reduced LT-HSC number at steady state and during regeneration. Reduced HSC repopulating capacity in a competitive setting. Reduced Hes1 and Hey1 in HSCs after endothelial deletion of Jag1	Poulos et al., 2013
VEcad-cre; Jag2fl/fl mice	Endothelial cells	Hes, Hey1	Unchanged LT-HSC number at steady state and reduced LT-HSC number during regeneration. Reduced capacity for Jag2ECKO mice HSCs to differentiate into T cells. Reduced Hey1 and increased Hes1 in HSCs after endothelial deletion of Jag2	Guo et al., 2017
Mx1-cre; RBPJfl/fl	Hematopoietic cells and stromal cells	miR-155	Notch blocks HSC proliferation in a cell-autonomous manner; Notch in the BM microenvironment inhibit the onset of MPN disease via repressing NF-kB signaling	Wang L. et al., 2014
RafVCC; Rbpjfl/fl mice RafVCC; Dll4fl/fl mice; RafVCC; Fbxw7 mice; RafVCC; NICD mice	Endothelial cells	Not reported	Enhanced Notch activity in endothelial cells increases the number of ephrin-B2^+^ ECs, Pdgfrb^+^ perivascular cells, cellular SCF levels and HSC cell number. Endothelial Notch reactivates HSC niches in aged mice	Kusumbe et al., 2016
Vecad-cre; Notch1fl/fl mice; Notch1^+^/deltaTAD	Global or endothelial cells	Hes1, Myc, Hey1, Dtx1, pTa, EphrinB2, Dtx1	Endothelial Notch1 activity promotes HSC regeneration by maintaining endothelial cell niche. Notch1 activity is required for CLP and T cell differentiation in a cell-autonomous manner	Shao et al., 2019
Thymocytes overexpressing Lfng	Thymocytes	Not reported	Notch1 activity in early thymocytes functions to suppress B cell differentiation; Lfng inhibits Notch1 activity	Koch et al., 2001
Bone marrow cell retroviral transduction of dnMAML	Hematopoietic cells	Not reported	dnMAML functions as a pan-Notch inhibitor. dnMAML inhibits T cell differentiation, promoted the emergence of B cells in thymus	Maillard et al., 2004
Mx1-cre; Notch1fl/fl mice; Mx1-cre; Jag1fl/fl mice	Hematopoietic cells and stromal cells	Not reported	Deletion of Jag1 using Mx1-cre does not impact LT-HSC cell number at steady state or during regeneration; Mice with Jag1 and Notch1 deletion regenerate their HSC normally	Mancini et al., 2005
dnMAML transduced human HSCs	Hematopoietic cells	Dtx1, Gata1	Notch signaling is required for *in vitro* expansion of human HSCs and delays the onset of multipotent progenitor cells	Benveniste et al., 2014
Notch1^−/−^ mice; Notch2^−/−^ mice	Global knockout mice	Not reported	Notch1 but not Notch2 is required for the emergence of HSC from hemogenic endothelium	Kumano et al., 2003
Mx1-cre; Rosa26<stop>ICD fl/fl mice; Mx1-cre; Notch1fl/fl; Notch2fl/fl	Hematopoietic cells and stromal cells	Hes1, Gata3, Dtx1, Hey1, Nrap	Notch2 gain of function enhances erythroid differentiation; Notch1, Notch2 loss of function decreases stress erythropoiesis	Oh et al., 2013
Vav1-cre; Ncstn fl/fl mice; Mx1-cre; Ncstn fl/fl mice; Mx1-cre; Ncstn fl/fl, N1IC mice	Hematopoietic cells and stromal cells	Not reported	Somatic Notch inactivation mutation was found in CMML patients. Deletion of gamma secretase component Ncstn lead to onset of CMML like disease and increase of myeloid gene signature in LKS cells	Klinakis et al., 2011
Mx1-cre; Pofut1 mice	Hematopoietic cells and stromal cells	Not reported	Hematopoietic cell and niche Pofut1 both contributed to loss of T cells and myeloid hyperplasia	Yao et al., 2011
MLL-AF9 Rosa wt/creERT2 mice; MLL-AF9, Rosa lsl-N2-IC/creERT2 mice; Ncstn^−/−^tet2^−/−^ mice	All cells in mice	HES1, HEY1, NRARP; Bcl2, Adamdec1, Itgax, Mmp9, Cd74	Notch is silenced in human AML samples; activation of Notch signaling lead to cell cycle arrest and apoptosis of AML cells; Combined Notch and Tet2 loss of function led to AML in mice	Lobry et al., 2013
Retroviral mediated overexpression of Notch receptors or Hes1	Human AML cells	Hes1	Notch1, Notch2 or Hes1 overexpression in AML inhibits their growth and survival; Notch inhibitor dnMAML enhances *in vivo* AML engraftment	Kannan et al., 2013
Immobilized Dll1	Human CD34^+^ cells	Not reported	Immobilized Dll1 increase the number of *ex vivo* expanded human CD34^+^ cells that repopulate NOD-SCID mice	Ohishi et al., 2002
Immobilized Dll1	Human CD34^+^ cells	Not reported	Immobilized Dll1 increased the number of *ex vivo* expanded human CD34^+^ cells in the presence of cytokines SCF, Il6, Flt3, Il-11, and inhibited myeloid differentiation	Varnum-Finney et al., 2003
Retroviral mediated overexpression of Hes1	Human CD34^+^ cells	Not reported	Hes1 overexpression preserves purified HSCs *in vitro* and enriches side population HSC *in vivo*	Kunisato et al., 2003
Immobilized Dll4	Human CD34^+^ cells	Not reported	Membrane-bound Delta-4 Notch ligand reduces the proliferative activity of primitive human hematopoietic CD34^+^ CD38low cells while maintaining their long term colony-initiating cell potential	Lauret et al., 2004
Immobilized Dll4	Human CD34^+^ cells	E2F4, Rb	The Notch Delta-4 ligand helps to maintain the quiescence and the short-term reconstitutive potential of hematopoietic progenitor cells	Catelain et al., 2014
Immobilized Dll1	Human CD34^+^ cells	Not reported	Immobilized Dll1 increased the number of expanded human CD34^+^ cells and results in engraftment and rapid myeloid reconstitution	Delaney et al., 2010
Endothelial-HSC coculture, Mx1-cre; Notch1, Notch2 mice	Mouse HSCs	Hes1	Endothelial cell promote the expansion of LT-HSCs. Notch signaling is required for efficient HSC expansion	Butler et al., 2010
Bone marrow stromal cells overexpressing Dll1-HSC coculture	Mouse HSCs	Not reported	Bone marrow stromal cells overexpressing Dll1 inhibits B cell differentiation and promotes T cells	Schmitt and Zúñiga-Pflücker, 2002
OP9-DL1-HSC coculture	Mouse HSCs	Not reported	Notch signaling is required for T cell differentiation at DN1 and DN2 stage. The fate decision between T cell and B cell happens before double negative stage	Schmitt et al., 2004
OP9-DL1-HSC coculture	Human CD34^+^ cells	Not reported	OP9 cells overexpressing Dll1 promote the differentiation of human CD34^+^ cells into T cells that can be activated *in vitro*	Motte-Mohs et al., 2005
Mouse Fibroblast overexpressing Dll4; human fibroblast overexpressing Dll4	Human CD34^+^ cells	Not reported	Mouse fibroblast, but not human fibroblast or NIH3T3 cells overexpressing Dll4 promote efficient differentiation of human CD34^+^ cells into T cells	Mohtashami et al., 2013
OP9-Dll1-HSC coculture; OP9-Dll4-HSC coculture	Mouse HSCs	Dtx1, Nrarp, Gata3	Dll4 maintains its ability to repress B cells and promote T cell differentiation even at lower dose	Mohtashami et al., 2010
OP9-Jag2-HSC coculture	Human CD34^+^ cells	Hes1, Dtx1, Nrarp, Notch3, Gata3, Tcf7	Jagged2 acts as a Delta-like Notch ligand during early hematopoietic cell fate decisions to favor T cell differentiation	Walle et al., 2011
Mx1-cre; RBPJfl/fl mice	Hematopoietic cells and stromal cells	Not reported	RBPJ disruption leads to increased mortality and sensitivity to total body irradiation. Mouse Dll1 infusion increased the myeloid cell numbers during regeneration	Chen et al., 2016
RafVCC; Dll4fl/fl mice; RafVCC; Dll1f/fl mice	Endothelial cells	Not reported	Reduction of Dll1 and Dll4 expression in BM endothelial cells following stress; endothelial Dll4 deletion did not reduce LT-HSC cell number but lead to myeloid skewing of LT-HSCs	Tikhonova et al., 2019
Nod-scid mice with T-ALL xenografts	N/A	Not reported	Endothelial cell expression of Dll4 correlated with increased tumor proliferation. Inhibiting Dll4 lead to tumor dormancy	Indraccolo et al., 2009
Anti-Notch neutralizing antibodies	B-ALL	Not reported	Notch3/4 signaling and Jagged1/2, Dll1 promote the survival of B-ALL cells	Kamdje and Krampera, 2011
Neutralizing antibodies for Dll4, Notch1, Notch2/3	T-ALL	Dtx1, Hes1	Dll4 is expressed in the microenvironment of NOD-SCID mice bearing T-ALL. Blocking Dll4 induces T-ALL death	Minuzzo et al., 2015
MMTV-cre; Mib1fl/fl mice; Mx1-cre; Mib1fl/fl mice	Hematopoietic cells and stromal cells	Not reported	Defective Notch activation in microenvironment leads to myeloproliferative disease	Kim et al., 2008
siRNA mediated reduction of Notch1 in endothelial cells; Vecad-cre, Notch^+/−^ mice	Endothelial cells	Hes1, Heyl	Endothelial NOTCH1 is suppressed by circulating lipids and antagonizes inflammation during atherosclerosis	Briot et al., 2015
Vecad-cre, Notch^+/−^ mice; human endothelial cell under shear stress	Endothelial cells	Not reported	NOTCH1 is atheroprotective and acts as a mechanosensor in adult arteries	Mack et al., 2017
ApoE/; RBPJflox/flox; Cdh5-CreERT	Endothelial cells	Hes1, Vcam1, Icam1, Nfkbia, Myd88	Endothelial Jag1-RBPJ signaling promotes inflammatory leucocyte recruitment and atherosclerosis	Nus et al., 2016
ICN1-T-ALL mice	Osteoblast cells	Dtx1, Hes1, Hey1, Notch2	Stromal Notch activation in ICN1 T-ALL marrow leads to suppression of osteoblastic cells and their HSC supporting functions	Wang et al., 2016

## Canonical and Non-Canonical Notch Signaling

**Figure 1:**
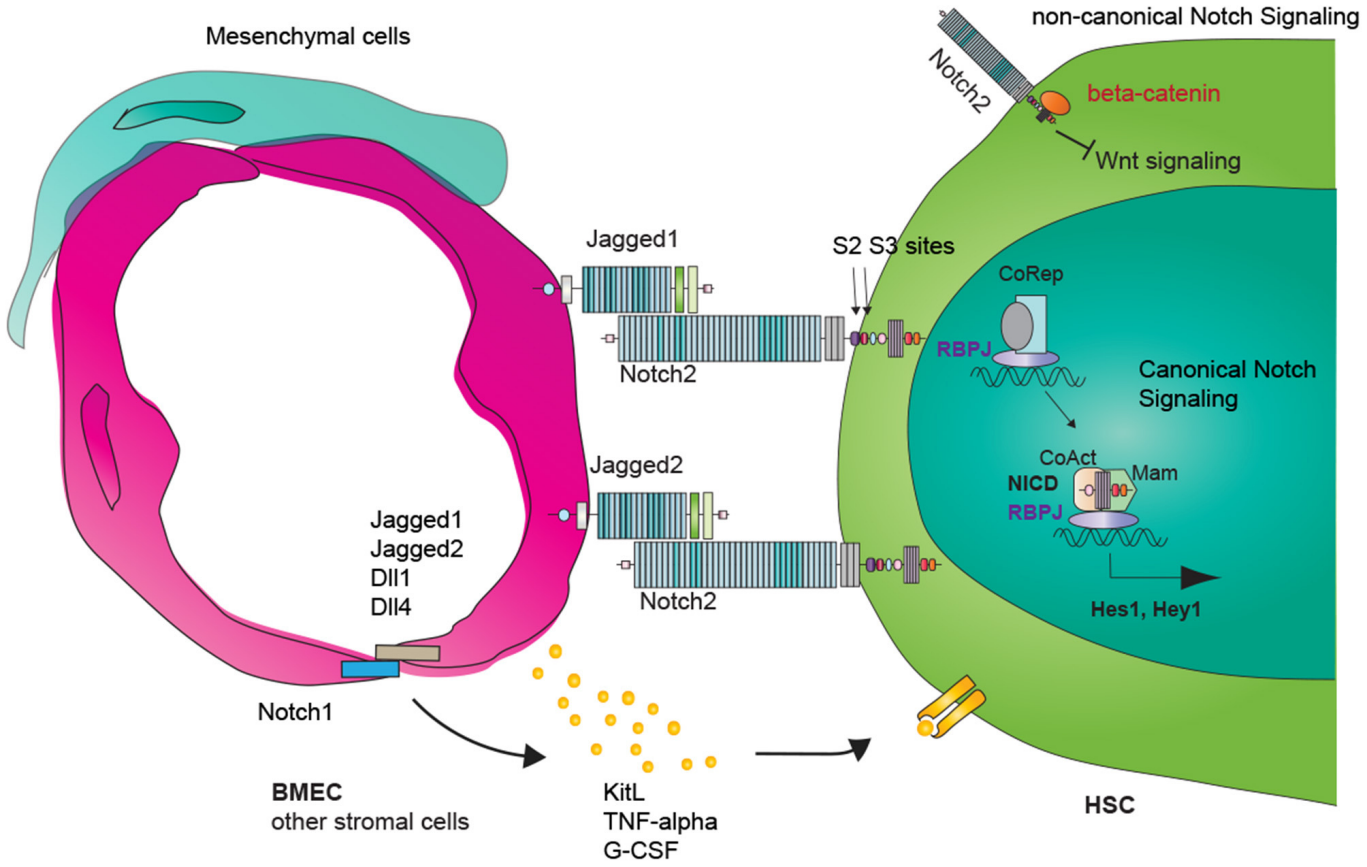
Vascular Notch regulation of adult hematopoietic stem cell (HSC) maintenance and regeneration. Canonical Notch signaling involves ligand and receptor binding, Notch receptor subsequent cleavages and release of the Notch intracellular domain (NICD). NICD translocates into the nucleus and turns on RBPJ/NICD-dependent transcription (canonical Notch signaling). RBPJ-independent NICD function is defined as non-canonical Notch signaling. Vascular endothelial cells express Notch ligands Jagged1, Jagged2, Dll1, Dll4, and Notch receptors. Vascular Notch ligand induces Notch downstream target expression within HSCs (cell-autonomous). Notch signaling within endothelial cells could lead to changes in inflammatory signaling and paracrine secretome, including TFN-alpha, GCSF, and KitL, which could then impact HSC behavior cell non-autonomous.

Canonical Notch signaling initiates when one of the five Notch ligands, Jagged-1, Jagged2, Delta-like 1 (Dll1), Dll3, or Dll4, binds with one of the four Notch receptors Notch1, Notch2, Notch3, or Notch4 (Fortini, 2009). The physical binding of ligand–receptor generates the mechanical strength to expose the Notch cleavage site S2 to disintegrin and metalloproteinase domain-containing protein 10 (ADAM10). Notch is subsequently cleaved at cleavage S3 by γ-secretase complex, a protein complex composed of presenilin, nicastrin, Aph-1, Pen-2, and others (Strooper et al., 1999; Meshorer and Misteli, 2005). The resulting Notch intracellular domain (NICD) translocates into the nucleus and dissociates the inhibitory complexes (corepressors, CoR, and histone deacetylase complex HDAC) bound with Recombination Signal Binding Protein for Immunoglobulin Kappa J Region (RBPJ). NICD/RBPJ then recruits the co-activator complexes mastermind like transcription coactivator (MAML), and initiates the transcription of Notch target genes. Besides the RBPJ-kappa-associated module (RAM) domain that binds RBPJ, the C-terminus of Notch contains an ankyrin repeat domain that interacts with other proteins, a transactivation domain, and a PEST domain that targets NICD for proteasomal degradation, providing a negative feedback for Notch signaling (Figure 1, right side).

The regulation of Notch signaling intensity takes place at multiple levels. Foremostly, Notch ligands and receptors are differentially expressed within specific biological contexts. Biochemical experiments using recombinant Notch ligands and receptors have demonstrated the ability of binding of each of the Notch ligands to each of the Notch receptors (Lindsell et al., 1995; Luo et al., 1997; Shimizu et al., 1999, 2000). However, Notch ligands can bind the same receptor with different intensities, inducing a distinct expression of Notch target genes (Andrawes et al., 2013; Nandagopal et al., 2018; Tveriakhina et al., 2018). Secondly, the post-translational modifications, such as glycosylation by Lunatic fringe (Lfng) or o-fucolysation by protein O-fucosyltransferase 1 (Pofut1) of the Notch receptors, alter their binding with Notch ligands. Thirdly, whether the ligands and the receptors are coexpressed on the same cell (cis-) or in neighboring cells (trans-) also influences the signaling outcomes (Benedito et al., 2009; Kopan and Ilagan, 2009; Palmer et al., 2014).

Whereas the canonical Notch signaling involves the ligand–receptor binding and NICD/RBPJ-dependent transcription of Notch downstream targets, non-canonical Notch signaling is either dependent or independent of ligand–receptor binding and does not involve NICD/RBPJ-mediated transcription regulation (Figure 1, right upper) (Andersen et al., 2012). A well-known example of non-canonical Notch functions is to regulate the Wnt/Catenin pathway. Notch binds and titrates the levels of the Wnt signaling pathway component, beta-catenin, and regulates stem cell fates (Hing et al., 1994; Fre et al., 2009).

## Notch Signaling in HSC Homeostasis and T Cell Differentiation

The interpretation of Notch signaling in HSC maintenance mainly comes from transgenic mouse models mutating/deleting key components of the Notch signaling pathway in hematopoietic cells or niche cells. Inducible inactivation of Notch1 led to T cell deficiency, which was transplantable when Notch1-deleted hematopoietic cells were engrafted into wild type (WT) recipients, demonstrating the cell autonomous role of Notch1 signaling in specifying T cell differentiation (Radtke et al., 1999). Notch1-deficient common lymphoid progenitors (CLPs) differentiated into B cells instead of T cells in the thymus (Wilson et al., 2001). Using Mx1-cre to induce the deletion of RBPJ, Han et al. also demonstrated the requirement of canonical Notch signaling in T cell differentiation. Several other studies have pointed out that Notch signaling components, including Lfng or MAML, could regulate T cell differentiation in the thymus (Koch et al., 2001; Maillard et al., 2004). It is therefore established that Notch signaling is required for T lineage specification. To briefly extend from normal homeostasis to hematopoietic malignancies, Notch1 gain-of-function mutation is found in about 50% of T-ALL patients. Notch1 mutation occurs within the heterodimerization domain, the juxtamembrane domain, or PEST domain and have been associated with Notch hyperactivation upon ligand engagement or reduced ubiquitination and degradation (Aifantis et al., 2008; Li and Boehmer, 2011; Radtke et al., 2013; Ntziachristos et al., 2014). This demonstrates the oncogenic role of Notch signaling in promoting T-ALL.

On the other hand, canonical Notch signaling is dispensable for adult HSC homeostasis. In a Mx1-cre; Jag1fl/fl (Jag1^−/−^) mouse model, deletion of Jagged1 did not alter the percentage or number of LT-HSCs in the BM during homeostasis (Mancini et al., 2005). After 5-flurouracil (5-FU)-induced myelosuppression, Jag1^−/−^ and Notch1^−/−^ combined deletion did not lead to the inferior recovery of LT-HSCs. Competitive transplantation experiments did not reveal a disadvantage of Jag1^−/−^ or Notch1^−/−^ hematopoietic cells in repopulating the WT mice, nor a disadvantage of Notch1^−/−^ HSCs in repopulating Jag1^−/−^ mice compared with WT cells into WT mice. Furthermore, HSCs with an inducible overexpression of dominant-negative MAML1 from Mx1-cre; dnMAML1 mice could reconstitute long term in CD45.1-irradiated mice without changing the HSC cell numbers in a competitive setting (Maillard et al., 2008). In the same study, the authors used a mouse model with Mx1-cre-mediated RBPJ deletion. After competitive transplantation, the percentage of total CD45.2^+^ cells derived from RBPJ^−/−^ cells remained the same as the percentage of CD45.2^+^ cells derived from WT cells; RBPJ^−/−^ donor cells demonstrated myeloid cells or pro-B cell bias at the expense of T cell deficiency (Maillard et al., 2008). These lines of evidence demonstrate that canonical Notch signaling is dispensable for adult HSC maintenance. Using Mx1-cre; RBPJ mice, Duarte et al. showed that canonical Notch signaling was dispensable for all stages of megakaryocyte, erythroid, and myeloid progenitor cells in homeostasis and regenerative hematopoiesis (Maillard and Pear, 2018). In human HSCs, dnMAML-mediated inhibition of Notch activity showed that Notch signaling is required for the *in vitro* expansion but not the *in vivo* reconstitution of HSCs (Benveniste et al., 2014). These lines of evidence underlies the notion that cell autonomous canonical Notch signaling is dispensable for adult HSC maintenance.

## Notch2 Signaling Is Necessary for HSC Regeneration

During myelosuppression triggered by sublethal irradiation or 5-FU administration, the rapid death of fast-cycling blood cells induces the cell cycle entry of quiescent HSCs. Perhaps such stress scenarios necessitate the tighter regulation imparted by Notch signaling to prevent HSC exhaustion (Varnum-Finney et al., 2011). This study demonstrates the requirement of Notch2 to promote HSC regeneration and block myeloid differentiation. The requirement of Notch2, but not Notch1, in adult HSC regeneration is in contrast to the requirement of Notch1 in endothelial-to-hematopoietic transition during embryonic development (Kumano et al., 2003), suggesting the temporal requirement of distinct Notch receptors, which could be explained by the relevant abundance of Notch2 compared with Notch1 expression. Oh et al. systemically mapped the expression of Notch receptors within the hematopoietic system; Notch2 is more abundantly expressed in adult HSCs compared with Notch1. The expression of Notch2 was confirmed by qRT-PCR in the studies by Klinakis et al., Poulos et al., and Guo et al. Notch1 plays a more important role in T cell differentiation. In the study of Oh et al., it has been shown that Notch2 and Notch1 are coexpressed within lymphoid-primed multipotent progenitor cells and CLPs; as the progenitor cells move toward T cell specification, Notch1 becomes the predominant receptor in double-negative T progenitor cells, corroborating with the finding that hematopoietic Notch1 deletion led to T cell deficiency (Radtke et al., 1999). These lines of evidence suggest the requirement of Notch receptors at specific biological contexts correlated with the relative abundance of receptor expression.

## Notch Signaling Represses Myeloid Differentiation and Promotes Erythroid Differentiation

Lines of evidence support the role of Notch signaling in repressing HSC myeloid differentiation. Competitive transplantation experiment using Mx1-cre; RBPJ^fl/fl^ (RBPJ^−/−^) cells showed that RBPJ repressed HSC myeloid differentiation into CD11b^+^Gr1^+^ cells (Maillard et al., 2008). Pofut1 catalyzes the transfer of fucose to the serine or threonine residues of epidermal growth factor-like (EGF) repeats within Notch receptors and is required for Notch signaling transduction; the conditional deletion of Pofut1 led to myeloid differentiation at the expense of T cells (Yao et al., 2011). Vav1-cre- or Mx1-cre-mediated deletion of gamma secretase component nicastrin (Ncstn) or deletion of Notch1, 2, and 3 (mainly Notch1 and 2) mimicked the CMML phenotypes in mice (Kalaitzidis and Armstrong, 2011; Klinakis et al., 2011; Lobry et al., 2013). Mx1-cre; Ncstn^−/−^ mice had increased numbers of granulocyte macrophage progenitor cells (GMPs); Ncstn^−/−^ LKS cells demonstrated a myeloid signature mimicking GMPs. Overexpression of Notch1, Notch2, or Hes1 could repress the myeloid gene signatures in Ncstn^−/−^ LKS cells. Notably, CMML patient BM samples, but not their normal tissues, demonstrated loss-of-function mutations in genes related to the Notch signaling pathway, including NOTCH2, gamma secretase components NCSTN and APH-1, as well as MAML1, suggesting the somatic origin of such mutations. In a subsequent study, it has been shown that Notch reactivation (especially Notch2) within the acute myeloid leukemia (AML)-initiating cells was sufficient to drive their cell cycle arrest, differentiation, and apoptosis without inducing T-ALL (Kannan et al., 2013; Lobry et al., 2013). Notch has thus been proven to serve as a tumor suppressor in myeloproliferative diseases, besides its oncogenic role in mediating T-ALL and certain solid cancers (Ntziachristos et al., 2014); Notch activation emerges as a promising approach to combat AML. On the other hand, Duarte et al. has demonstrated that Notch signaling is dispensable for homeostasis or regenerative myelopoiesis. We will discuss these conflicts toward the end of this subsection.

Conflicting evidence also exists regarding whether Notch regulates erythroid differentiation or not. Oh et al. demonstrated that Notch signaling was required for the proper differentiation of erythroid lineages during sublethal irradiation or phenylhydrazine-triggered stress erythropoiesis using Mx1-cre; Notch1^−/−^, Notch2^−/−^ mice, and Vav1-cre; Ncstn^−/−^ mouse models. The Mx1-cre-mediated overexpression of Notch2 intracellular domain ICN2 led to an increased number of CFU-Es in the BM and a reduction of platelets in the peripheral blood. This demonstrated the role of Notch2 signaling in promoting erythroid lineage differentiation and repressing platelet differentiation. The discrepancy of Notch signaling regulation of myeloid and erythroid specification described in Oh et al. study and Duarte et al. study could be related to the different molecules deleted; Oh et al. used Mx1-cre; Notch1^−/−^ and Notch2^−/−^ mice that depleted the Notch receptors and Vav1-cre; Ncstn^−/−^ mice that deplete one of the gamma-secretase inhibitor components, Ncstn. On the other hand, Duarte et al. used the Mx1-cre; RBPJ^−/−^ model that depleted the canonical Notch signaling component RBPJ to show unchanged myelopoiesis, erythropoiesis, or megakaryopoiesis. It is possible that RBPJ-independent, non-canonical Notch1 and Notch2 signaling regulate myeloid or erythroid differentiation.

A detailed examination of RBPJ or NICD binding sites across the genome might help interpret the contradictory results. In an effort to map the RBPJ binding sites in the absence or presence of Notch signaling, (Castel et al., 2013) has shown that, within C2C12 myoblasts, there are two classes of RBPJ binding sites: (1) the dynamic binding sites that RBPJ/NICD co-binds only in the presence of active Notch signaling together with transcription coactivator p300 and (2) static RBPJ binding sites where RBPJ recruits neither NICD nor p300. In T-ALL, the dynamic RBPJ binding sites are enriched with Notch1 signal; there are also many sites that only bind with RBPJ in human TALL (Wang et al., 2011). At the dynamically regulated sites, Notch1 interacts with super-enhancers in T-ALL to initiate transcription (Wang H. et al., 2014). RBPJ has binding sites that are independent of NICD binding. Deletion of RBPJ therefore not only aborts the canonical Notch signaling but also aborts its regulatory role irrelevant to Notch signaling. Moreover, Notch1/2 may function in both an RBPJ-dependent manner and an RBPJ-independent manner (Andersen et al., 2012). These may underlie the observed phenotypic differences between myeloid and erythroid lineage differentiation in the above-mentioned two studies. We conclude that Notch signaling blocks myeloid differentiation and promotes stress erythropoiesis, likely through the non-canonical signaling branch.

## Notch Signaling Enhances *ex vivo* HSPC Expansion and T Cell Differentiation

A combination of Notch signaling and cytokine effects induces the proliferation of hematopoietic progenitor cells. Immobilized Dll1 induced Notch signaling as shown by Hes1 transactivation and was used to expand human cord blood HSCs *ex vivo* (Ohishi et al., 2002; Varnum-Finney et al., 2003). Hes1 preserved purified HSCs *ex vivo* and accumulates side populations (Kunisato et al., 2003). Dll4 has been demonstrated to preserve the stemness of HSCs but inhibited the growth of hematopoietic progenitor cells *in vivo* (Lauret et al., 2004). Dll4 retained the quiescence of HSCs and preserved the repopulating capacities of short-term HSCs cultured *ex vivo* (Catelain et al., 2014). Delaney et al. further developed a clinically relevant platform for the *ex vivo* expansion of human CD34^+^ cord blood progenitors using immobilized Dll1, which led to marked increases in the absolute numbers of expanded HSPCs and rapid myeloid reconstitution following HSPC transplantation. These lines of evidence demonstrated the requirement of robust Notch activation in promoting HSPC *ex vivo* expansion.

Notch signaling is also essential for the expansion of mouse or human HSCs in a vascular niche (Butler et al., 2010; Benveniste et al., 2014; Poulos et al., 2015). Within the endothelium–HSC coculture system, the contact between HSC and endothelial cells was required for optimal expansion; the cocultured HSPCs increased their Notch activity overtime. Notch1, 2^−/−^ HSCs did not expand efficiently but rather underwent exhaustion. This evidence demonstrates the requirement of Notch1, 2 signaling for effective HSC expansion without premature exhaustion. The vascular niche has also been used to effectively expand human CD34^+^ cord blood stem cells (Butler et al., 2012).

Notch promoted HSC differentiation into T cells on an OP9-Dll1 feeder (Weekx et al., 2000; Schmitt and Zúñiga-Pflücker, 2002; Schmitt et al., 2004; Motte-Mohs et al., 2005). Dll4 also induced the differentiation of HSPCs into T cells when overexpressed in fibroblasts (Mohtashami et al., 2013). When overexpressed in OP9 cells, Dll1 and Dll4 elicited different Notch signals and influenced the lymphomyeloid lineages, suggesting ligand-specific roles (Mohtashami et al., 2010). Within *ex vivo* coculture setting, Jagged2 acted like a delta-like ligand to promote the differentiation of early T cell progenitors, albeit binding with Notch1 at a lower affinity (Walle et al., 2011). These lines of evidence demonstrate the requirement of Notch signaling to maintain HSC *ex vivo* expansion or to promote T lymphopoiesis. At an *in vitro* setting, Notch also specified the megakaryocytic lineages. OP9-Dll1 promoted the differentiation of mouse LKS or human CD34^+^ HSCs into CD41^+^ megakaryocytes, which was dependent on canonical Notch signaling. HSCs overexpressing Notch4 intracellular domain differentiated into megakaryocyte at the expense of GMPs or erythroid lineages when transplanted into irradiated mice (Mercher et al., 2008). The differences in the requirement of Notch signaling between *in vitro* and *in vivo* settings could be due to the *in vivo* niche filling mechanisms, wherein a plethora of other signaling events in the microenvironment compensates for the loss of canonical Notch signaling. Nonetheless, for the consideration of engineering durable vascular niche or stromal niche to expand *bona fide* HSCs or for T cell differentiation, the maintenance of Notch signaling is essential.

## Vascular Notch Signaling in HSC Homeostasis and Regeneration: Cell Autonomous or Non-Autonomous

### Endothelial Cell-Specific Deletion of Notch Ligands: Cell Autonomous

Our understanding of Notch signaling contribution to HSC maintenance comes from the deconvolution of niche cell-derived Notch ligands and their dynamic changes following stress. Endothelial cell-derived Notch signaling contributes to homeostatic and regenerative hematopoiesis (Poulos et al., 2013; Guo et al., 2017). In VEcad-cre; Jag1fl/fl mice where Jag1 was specifically knocked out from endothelial cells (termed as Jag1ECKO mice), the number of phenotypic LT-HSC was reduced at steady state. The repopulating capacity of HSCs was diminished as assessed by competitive transplantation assay. HSC also regenerated less efficiently following myelosuppression, such as sublethal irradiation and serial 5-FU injections (Poulos et al., 2013). The requirement of endothelial cell Jagged1 in maintaining HSC reconstitution is in contrast to the dispensable role of Jagged1-mediated Notch signaling in stress homeostasis in an earlier Mx1-cre; Jag1fl/fl model (Mancini et al., 2005). Mx1-cre deleted Jag1 from hematopoietic cells, but not endothelial cells, as confirmed by Poulos et al.; it is possible that although cell-autonomous Jagged1 is dispensable for HSC maintenance, endothelial cell-derived Jagged1 is necessary for HSC homeostasis and regeneration. Endothelial Jagged1 induced the activation of Hes1 and Hey within HSCs (Figure 1, middle); HSCs harboring a Hes1-GFP reporter had reduced Hes1 signals when transplanted into Jag1ECKO mice (Poulos et al., 2013). Chen et al. also demonstrated the requirement of Notch signaling during HSC regeneration. Disruption of Notch signaling aggregated irradiation-induced BM injury, which was ameliorated by a soluble Dll1 ligand through Csf2rb2 upregulation.

Using mice with endothelial cell-specific deletion of Jagged2 (VEcad-cre; Jag2fl/fl mice, termed as Jag2ECKO mice), Guo et al. showed that there was an increase of total hematopoietic cell numbers in the BM of Jag2ECKO mice. HSCs from Jag2ECKO mice repopulated into irradiated mice at the same level compared with WT HSCs in a competitive setting (Guo et al., 2017). Ligand expression levels within endothelial cells could underlie the differential requirement of Jagged1 vs. Jagged2 during homeostasis. Jagged1 is expressed at a higher level compared with Jagged2 within BMECs at a steady state (Tikhonova et al., 2019). In regenerative setting, endothelial Jagged2 promoted HSC recovery by maintaining their quiescence. HSCs from Jag2ECKO mice had a reduced capacity to differentiate into T cells in a competitive transplantation setting; there was an increased percentage of B cells in the peripheral blood of aged Jag2ECKO mice. Therefore, endothelial Jagged2 favors HSC differentiation into T cells over B cells, which corroborated with earlier findings that RBPJ-dependent Notch1 signaling specifies T cell lineages over B cell lineages (Han et al., 2002) and suggested the direct signaling between endothelial-Jagged2 with HSCs that involves canonical Notch signaling RBPJ. Endothelial Jagged2 induces Hey1 but blocks Hes1 signaling in HSCs, which is in contrast to both Hes1 and Hey1 activation by endothelial Jagged1, suggesting ligand-specific roles in triggering downstream Notch targets in HSCs. Comparing with the earlier report that canonical Notch signaling is dispensable for adult HSC homeostasis, the experimental efforts described in this section highlighted the critical roles of endothelial Notch ligands in HSC homeostasis and regeneration.

Tikhonova et al. pinpointed the enrichment of Notch ligand Dll1 and Dll4 within BMECs, but not other niche components, using single-cell RNA-seq of BM vascular or stromal cells, coupled with Dll1, Dll4, and Jag1 reporter mice to monitor the Notch ligand expression. Following stress, endothelial cells downregulated Dll1 or Dll4, which correlated with the increased percentage of myeloid cells at this stage. Mice with endothelial deletion of Dll4 had increased numbers of GMPs and reduced numbers of CLPs; HSCs in Dll4-ECKO mice were not reduced in number but showed myeloid gene enrichment compared with WT HSCs. In contrast, deletion of endothelial Dll1 did not affect early lineage priming of hematopoietic progenitors. This demonstrates the distinct functions of endothelial Dll4 and Dll1 in regulating HSC maintenance and early lineage differentiation. It was previously shown that Dll4 binds with Notch1 at a stronger binding affinity and triggers different downstream signals compared with Dll1 (Andrawes et al., 2013; Nandagopal et al., 2018; Tveriakhina et al., 2018), which could be contributing to the observed differences. The outstanding question remains whether endothelial Dll4 blocks HSC myeloid priming *via* direct engagement with HSC Notch receptors and/or indirect effect on endothelial cells.

For hematopoietic malignancies, microenvironment-derived Notch signaling regulates disease progression. The crosstalk between Notch3 on T-ALL and bone marrow endothelial Dll4 promoted the expansion of T-ALL; during angiogenesis, endothelial cell tip cells overexpress Dll4 following VEGF induction which activated Notch3. Silencing Notch3 reduced the proliferation of T-ALLs (Indraccolo et al., 2009). Notch3- and Notch4-mediated signaling also promoted the survival of B-ALL that were in contact with bone marrow mesenchymal stromal cells (Kamdje and Krampera, 2011; Kamdje et al., 2011). Dll4 was expressed in the BM microenvironment of T-ALL patients and NOD-SCID mice bearing the T-ALL xenograft; Dll4 blockage impaired T-ALL growth in NOD-SCID mice and increased leukemia cell apoptosis (Minuzzo et al., 2015). Therefore, inhibiting the interaction of the microenvironment-derived Notch ligands with T-ALL has the potential to block their growth. On other hand, for CMML disease wherein Notch plays a tumor suppressor role, the increase of Notch receptors within tumor cells or overexpression of Notch ligands in the microenvironment could serve as a treatment paradigm.

### Niche Cell-Specific Deletion of Notch Receptors: Cell Non-autonomous

Modulating Notch signaling in endothelial cells or other niche cells also leads to changes in HSC maintenance and lineage differentiation. When Mindbomb-1 (Mib1), an essential gene for Notch ligand endocytosis, was deleted (Mx1-cre; Mib1^−/−^ mice or MMTV-cre, Mib1^−/−^1 mice), the mice developed *de novo* myeloproliferative neoplasm (MPN), which is attributable to the loss of Mib1 from the bone marrow microenvironment (Kim et al., 2008). Conditional overexpression of ICN1 within the Mib1 null bone marrow microenvironment suppressed the development of MPN (Kim et al., 2008). In another study, Wang L. et al. demonstrated that the myeloid bias of HSCs in RBPJ deletion mice (Mx1-cre; RBPJ fl/fl) was due to the RBPJ loss within the BM niche. Transplantation of WT hematopoietic cells into RBPJ^−/−^ mice, but not transplantation of RBPJ^−/−^ hematopoietic cells into WT mice, induced the onset of MPN, with increased numbers of GMPs in the BM and spleen. Furthermore, the MPN phenotypes, when RBPJ^−/−^ HSCs were transplanted into RBPJ^−/−^ mice, were more pronounced compared with when WT HSCs were transplanted into RBPJ^−/−^ mice, suggesting the contribution of cell autonomous Notch signaling in repressing MPNs, when there is also a disturbance of Notch signaling in the microenvironment. Mechanistically, RBPJ repressed miR-155, which repressed kB-Ras1 (NKIRAS1), a repressor of the NF-KB signaling pathway; deleting RBPJ in the niche therefore reduced NKIRAS1 expression and enhanced the NF-KB signaling, which correlated with MPN onset (Figure 1, left).

In a recent review by Mack et al., Notch regulation of endothelial cell inflammation was also summarized (Mack and Iruela-Arispe, 2018). On the one hand, Notch1 signaling reduces endothelial cell inflammation. High-fat diet reduced endothelial Notch1 levels *in vivo* (Briot et al., 2015). Notch1 expression is increased under atheroprotective flow; Notch1 loss resulted in atherosclerosis in a mouse model of hypercholesterolemia (Mack et al., 2017). On the other hand, Jagged1-RBPJ signaling promoted vascular inflammation through NF-kB and VCAM1 expression (Nus et al., 2016). The differences between Notch1- and Jag1-mediated endothelial cell inflammation could be mediated by the presence of other Notch ligands in endothelial cells. Nonetheless, it is possible that the niche Notch signaling regulation of HSC regeneration is partially due to the changes of inflammatory signaling within endothelial cells. Further studies are needed to clarify this notion.

During aging, Notch activity within endothelial cells is reduced in the BM microenvironment. Increased Notch activity by way of endothelial cell-specific overexpression of ICN1 or endothelial cell-specific deletion of Fbxw7 increased the types of endothelial cells suitable as HSC niche as well as HSC number (Kusumbe et al., 2016). Specifically, endothelial cell overexpression of Notch signaling led to increases in CD31-positive capillaries and PDGFRβ-positive perivascular cells, arteriole formation, and an elevation of cellular stem cell factor levels (Figure 1, left). In contrast, endothelial cell-specific deletion of RBPJ or Dll4 led to HSC reduction and myeloid biased differentiation, corroborating the findings within the previously described studies (Wang L. et al., 2014; Tikhonova et al., 2019) and highlighting the vascular Notch signaling in HSC maintenance and differentiation. For hematopoietic malignancies, there is an increase of Notch signaling in the bone marrow stromal cells in a mouse model of T-ALL (hematopoietic overexpression of ICN1); blocking the aberrantly active notch signaling within the bone marrow stroma cells rescued the malignant phenotypes associated with T-ALL, such as attenuated HSPC cycling, inadequate osteoblast differentiation, and thrombocytopenia (Wang et al., 2016).

Taken together, for the consideration of niche-derived Notch ligands in HSC function, it needs to be confirmed whether the Notch signaling components in the receiving HSCs have been changed and whether the changed Notch targets lead to the observed phenotypes. Due to the limitations of antibodies against NICD or RBPJ (Castel et al., 2013) and especially the small numbers of primary HSCs, it remains challenging to perform chromatin immunoprecipitation followed by sequencing (ChIP-seq) to identify the genomewide direct Notch targets within each biological context. Alternatively, changes in Notch signaling in endothelial cells could lead to changes in inflammatory pathway or stem cell factor secretion, which then crosstalk with HSC to affect their fate.

## Notch Signaling in T Cell Function in Tumor Microenvironment

We also herein include a brief introduction of the role of vascular Notch signaling in T cell function in the tumor microenvironment (TME), which fits within the scope of stress lymphopoiesis. The role of Notch signaling in regulating T cell differentiation, polarization, and function in diseases, such as graft vs. host disease and atherosclerosis has been extensively reviewed elsewhere (Brandstadter and Maillard, 2019; Sega et al., 2019); here we highlighted an area where vascular Notch ligand contribution of T cell function is lacking.

The TME comprises all structures recruited to the tumor sites, including cancer cells, immune cells, tumor vasculature, and the extracellular matrix. Each compartment within the TME expresses a unique set of Notch receptors and ligands and interacts with its neighboring structures, collectively contributing to tumor progression *via* autocrine and/or paracrine signaling (Meurette and Mehlen, 2018). In addition to its role in developmental T lymphopoiesis, mounting lines of evidence demonstrate that the Notch pathway is involved in the regulation of tumor angiogenesis, T cell differentiation, cytokine secretion, and immune response in solid tumors (Cho et al., 2009; Sierra et al., 2014, 2017; Colombo et al., 2016; Kayamori et al., 2016). However, the mechanism underlying Notch-mediated anti-tumor immune response remains elusive. This section is dedicated to review the involvement of Notch signaling in T cell immunity, particularly CD8^+^ T cell activation, effector-memory T cell differentiation, and regulatory T (Treg) cell recruitment, yet inconsistent results of Notch's function in T cell development have been reported (Table 2). We hope that the information discussed here could direct future efforts toward the understanding of how the Notch pathway within the vascular endothelial cells contributes to T cell immunity in solid tumors.

**Table 2:** Notch signaling in T-cell function within TME.

**Study model**	**Target cells**	**Notch target**	**Role/impact**	**Reference**
Notch1-knockdown mice, γ-scretase inhibitor (GSI)-treated CD8+ T cells	CD8+ T cells	Eomesodermin	Cytotoxic T lymphocyte activity	Cho et al., 2009
Notch1-knockdown mice, γ-scretase inhibitor (GSI)-treated CD8+ T cells	CD8+ T cells	Granzyme B		Cho et al., 2009; Kuijk et al., 2013
T-bet-KO naive CD4+ T cells	CD4+ T cells	Ifng	Integrate multiple signaling inputs to orchestrate T helper cell polarization	Bailis et al., 2013
GSI (or Dll4)-treated CD8+ T cells	CD8+ T cells	IFNγ	Regulate CD8+ T-cell activation, proliferation, and differentiation into functional effector cells	Kuijk et al., 2013
Naive CD4+ T cells	CD4+ T cells		Regulate cytokine production	Bailis et al., 2013
Notch1/2-KO effector CD8+ T cells	CD8+ T cells	IL-2 receptor α chain	Promote differentiation of effector CD8+ T cells	Backer et al., 2014
Naive CD4+ T cells	CD4+ T cells	IL-4	Regulate CD4+ T-cell diffrentiation into Th1 or Th2 cells	Amsen et al., 2004; Bailis et al., 2013
Notch1-knockdown mice, γ-scretase inhibitor (GSI)-treated CD8+ T cells	CD8+ T cells	Perforin		Cho et al., 2009
RBPJ-deficient CD4+ memory T cells	CD4+ T cells	RBPJ	Memory CD4+ T-cell maintenance	Maekawa et al., 2015
Naive CD4+ T cells	CD4+ T cells	Rorγt	Engage CD4+ T cells differentiation into Th17 cells	Bailis et al., 2013
GSI-treated CD4+ T cells	CD4+ T cells	T-bet	Regulate CD4+ T-cell diffrentiation into Th1 or Th2 cells	Minter et al., 2005
Notch1-depleted WM266-4 cells	WM266-4 cells	Soluble N-ethylmaleimide–sensitive factor attachment protein (SNAP) 23	Suppress MDSC migration to tumor sites	López-López et al., 2020
CD4+ T cells from Dll1-treated lung carcinoma tumor-bearing mice	CD4+ T cells	Stat1/2	Promote T-cell differentiation into Th1 cells	Biktasova et al., 2015

### CD8^+^ T Cells Notch Up Antitumor Activity

Tumor-specific cytotoxic T lymphocytes have been shown to be effective in eliminating cancer cells; however, the antitumor efficacy of CD8^+^ T cells easily waned due to the immune exhaustion of TME. To overcome the immunosuppressive states in TME, approaches to rejuvenate cytotoxic T lymphocytes have been sought to re-establish the antitumor activity. Inconsistent results in regard to Notch's role in T cell differentiation have been reported: Notch can redirect CD8^+^ T cells toward memory precursor cells, which are long-lived and can rapidly release multiple cytokines upon the presence of antigens to control invasion, though another study reported that Notch can be an essential hub to promote differentiation into terminal effector T cells, which are commonly short-lived due to their terminally differentiated status (Cho et al., 2009; Backer et al., 2014; Maekawa et al., 2015). Recently, the mechanism that Notch signaling utilizes to convert activated T cells to stem cell-like memory T cells is discovered. This conversion is facilitated by the mitochondria biogenesis and fatty acid synthesis induced by Notch signaling (Kondo et al., 2017, 2020). These cells can respond to antigen re-stimulation with great expanding and self-renewal potential, suggesting a possible sustained efficacy of the stem cell-like memory T cells. Such findings may help to improve the sustainability of adoptive T cell transfer and inform future applications in oncology therapy.

Regardless of whether Notch signaling promotes T cells into effector cells or memory cells, Notch2 but not Notch1 signaling has been demonstrated to be essential for potent anti-tumor immunity (Sugimoto et al., 2010). Using E8I-cre that specifically targeted CD8^+^ T cells, it was shown that E8Icre; Notch2fl/fl mice died faster than E8Icre; Notch1fl/fl after inoculation of ovalbumin-expressing thymoma; treatment with Notch2 agonist antibody or infusing dendritic cells overexpressing Dll1 further boosted the anti-tumor CD8^+^ T cell responses. This demonstrated the feasibility of overexpressing Notch2 to enhance cytotoxic responses in activated antigen-specific CD8^+^ T cells (Sugimoto et al., 2010; Sierra et al., 2014). The transgenic expression of NICD in CD8^+^ T cells could prevent themselves from the tumor-induced T cell tolerance, suggesting that the Notch-mediated pathway can promote the cytotoxic CD8^+^ T cell function in tumors.

### “NO” Notch in an Exhausted TME

Myeloid-derived suppressor cells (MDSCs) have been shown to dampen T cell-mediated anti-tumor immunity. Preferential expression of Jagged1 and Jagged2 ligands, but not Dll1 and Dll4, was detected in tumor-infiltrating MDSCs (Hanson et al., 2009; Sierra et al., 2017). A recent study showed that systemic anti-Jagged therapy could recruit CD8^+^ T cells into tumors to overcome MDSC-mediated immune suppression (Sierra et al., 2017). However, the endothelial cell-derived Notch ligand, Jagged1, enhanced the effector function of CD4^+^ T cells by upregulating the Notch-dependent transcription factor, T-bet, and the effector molecule, IFNγ, in a model of vasculitis (Wen et al., 2017). The expression of Notch ligands by vascular endothelial cells in TME and whether they contribute to anti-tumor immunity warrant further investigation. In a mouse model of lung cancer, activation of Dll1-mediated Notch signaling limited tumor angiogenesis as well as tumor progression; it also enhanced the efficacy of anti-EGFR targeted therapy, highlighting the therapeutic potential for Notch signaling pathway given its effects in shaping the TME, especially in enhancing anti-tumor T cell immunity (Biktasova et al., 2015). This is in line with an earlier study that selective stimulation of Dll1-Notch signaling in T cells rescued T cell function and inhibited tumor growth (Huang et al., 2011).

Intriguingly, depletion of Notch1 in cancer cells led to a decrease in Treg cells detected in the TME, suggesting the immune-suppressive aspects of tumor-associated Notch1 receptor (Qiu et al., 2018). Inhibition of tumor-associated Notch1 reduced the number of regulatory T cells and MDSCs in the TME but not in the peripheral organs, such as the spleen, in a CCL5-dependent fashion (López-López et al., 2020). In human melanoma cells, Notch1-depletion was associated with a decrease in the expression of SNAP23, together diminishing the secretion of CCL5 as well as sensitizing tumors to immune checkpoint inhibitors.

Moreover, MDSCs suppress Notch1 and Notch2 expression in activated CD8^+^ T cells in a nitric oxide (NO)-dependent manner. Blockade of NO synthase could restore the level of Notch1 and Notch2 in T cells as well as eliminate the MDSC-mediated immune suppression to re-establish anti-tumor responses (Raber et al., 2014). NO could promote vasculature growth and endothelial cell migration through cGMP-mediated pathways, collectively modulating tumor angiogenesis and growth. Though the crosstalk between NO and Notch signaling in tumor vasculature has yet to be illustrated, a better understanding of NO's role in Notch-mediated pathways in the TME may warrant novel Notch-directed cancer strategies (Jenkins et al., 1995; Jadeski et al., 2003). An example of Notch's interaction with the immune-modulatory pathway is the crosstalk between Notch and VEGF. Initially designed as an anti-angiogenesis therapy, anti-VEGF treatment was recently shown to have immune-modulatory effects by enhancing the anti-tumor activity of CD8^+^ T cells (Almeida et al., 2020). Taken together, the interplay between T cells and tumor vasculature as well as the involvement of Notch signaling in such network merits further investigation to shed light on next-generation anti-cancer strategies.

## Discussion

Within the hematopoietic systems, Notch influences HSC regeneration, differentiation, and hematopoietic malignancy transformation by regulating ligand–receptor interactions or non-canonical signaling events. The bone marrow microenvironment-derived Notch ligand and/or the Notch signaling within the niche cells crosstalk with HSCs or leukemia cells to influence their behavior. Urgent questions in the field include the mapping of the downstream notch targets within HSCs and designing the next-generation Notch ligand/receptor-based blocking reagents or agonists for cell-specific Notch signaling enhancement or blockade.

## Author Contributions

PG and CH devised the scope of the review and wrote the manuscript. PG wrote the involvement of vascular Notch signaling in stress hematopoiesis. CH wrote the section about Notch signaling involved in the T cell immunity in tumors. CAP and DY offered the critical insights into the role of Notch signaling in T cell immunity in solid tumor. GY helped polish the manuscript. All authors contributed to the article and approved the submitted version.

## Conflict of Interest

The authors declare that the research was conducted in the absence of any commercial or financial relationships that could be construed as a potential conflict of interest.
